# Cancer Vaccines, Treatment of the Future: With Emphasis on HER2-Positive Breast Cancer

**DOI:** 10.3390/ijms22020779

**Published:** 2021-01-14

**Authors:** Sandeep Pallerla, Ata ur Rahman Mohammed Abdul, Jill Comeau, Seetharama Jois

**Affiliations:** 1 School of Pharmaceutical and Toxicological Sciences and School of Clinical Sciences, College of Pharmacy, University of Louisiana Monroe, Monroe, LA 71201, USA; sandeep.pallerla1@gmail.com (S.P.); comeau@ulm.edu (J.C.); 2 Independent Researcher, Wharton, NJ 07885, USA; rahman.nam@gmail.com

**Keywords:** vaccine, breast cancer, HER2, therapeutic vaccine, cell-based vaccine, DNA-based vaccine

## Abstract

Breast cancer is one of the leading causes of death in women. With improvements in early-stage diagnosis and targeted therapies, there has been an improvement in the overall survival rate in breast cancer over the past decade. Despite the development of targeted therapies, tyrosine kinase inhibitors, as well as monoclonal antibodies and their toxin conjugates, all metastatic tumors develop resistance, and nearly one-third of HER2+ breast cancer patients develop resistance to all these therapies. Although antibody therapy has shown promising results in breast cancer patients, passive immunotherapy approaches have limitations and need continuous administration over a long period. Vaccine therapy introduces antigens that act on cancer cells causing prolonged activation of the immune system. In particular, cancer relapse could be avoided due to the presence of a longer period of immunological memory with an effective vaccine that can protect against various tumor antigens. Cancer vaccines are broadly classified as preventive and therapeutic. Preventive vaccines are used to ward off any future infections and therapeutic vaccines are used to treat a person with active disease. In this article, we provided details about the tumor environment, different types of vaccines, their advantages and disadvantages, and the current status of various vaccine candidates with a focus on vaccines for breast cancer. Current data indicate that therapeutic vaccines themselves have limitations in terms of efficacy and are used in combination with other chemotherapeutic or targeting agents. The majority of breast cancer vaccines are undergoing clinical trials and the next decade will see the fruitfulness of breast cancer vaccine therapy.

## 1. Introduction

Cancer is the second most common cause of death worldwide. According to the WHO, in 2018, approximately 9.6 million people died from cancer. Among the different types of cancers, lung, breast, prostate, and colorectal cancers are the most common. Each of these cancers contain molecularly defined subtypes and therefore vary in terms of incidence and prognosis. Breast cancer is the second most common reason for cancer-related fatalities in the United States in spite of the various recent improvements in diagnosis, prognosis, and treatment [1,2]. In the United States, approximately one in eight women during their lifetime will develop invasive breast cancer. Based on the data published in 2020, it is projected that about 276,480 women and 2000 men will be diagnosed with invasive breast cancer, and approximately 48,530 new cases of non-invasive (in situ) breast cancer will occur [3]. Breast cancer is defined as asymmetrical growth and proliferation of cells in the breast tissue [4]. In the last few decades, there has been an appreciable improvement in the treatment of breast cancer patients; however, there is a need to develop new, effective treatment strategies with minimal adverse effects.

The four major molecular subtypes of breast cancer include luminal A, luminal B, HER2-enriched, and basal-like triple-negative. This is determined by the expression of hormones (both estrogen and progesterone) and HER2 receptors. Triple-negative breast cancer (TNBC) is a condition where patients do not express the genes for estrogen receptor (ER), progesterone receptor (PR), and Her2/neu, thus making it difficult to treat TNBC patients. Along with HER2-enriched cancers, there is a subset of luminal B breast cancers that has HER2 overexpression [5,6,7]. HER2 belongs to the EGFR family receptors that play a crucial role in the pathogenesis of various cancers such as lung, breast, ovarian, and renal cancer [8]. The EGFR family includes 4 types of receptors; HER1 (EGFR), HER2, HER3, and HER4. Amongst these four receptors, HER2 receptors are overexpressed in different types of cancers. In about 20 to 30% of breast cancer patients, HER2 receptors are found to be overexpressed [9]. The HER2 overexpression is associated with enhanced tumor growth, poorer response to traditional chemotherapy, and overall decreased survival [10]; thus, researchers have focused on the development of HER2-based targeted therapies.

Treatment of HER2-positive breast cancer with chemotherapeutic agents alone elicited a poor response [11,12,13,14,15]. The discovery of tumor-associated antigens (TAA) has facilitated the emergence of immunotherapy. Immunotherapy with respect to cancer can be defined as the interference of the immune system for the mitigation of cancers [16]. Monoclonal antibodies that have anti-tumor properties were developed against the HER2 receptor. The intervention of the tumor growth via monoclonal antibodies falls under the category of passive immunity [17,18]. Trastuzumab was the first FDA-approved monoclonal antibody recommended for treating HER2-positive metastatic breast cancer. It causes anti-tumor effects through various mechanisms such as induction of apoptosis, induction of cell cycle arrest, antibody-dependent cell-mediated cytotoxicity (ADCC), inhibition of HER2 extracellular domain shedding, and inhibition of downstream signal transduction pathways [19,20,21].

Additionally, monoclonal antibodies and their conjugates such as pertuzumab, trastuzumab emtansine (T-DM1), and fam-trastuzumab deruxtecan were also approved by the FDA for treating HER2-positive breast cancer patients [22]. In the EMILIA study, T-DM1 exhibited improved survival for the second-line treatment of metastatic HER2-positive breast cancer compared to the existing standard therapy, capecitabine with lapatinib, a HER2 tyrosine kinase inhibitor [23]. T-DM1, compared to trastuzumab, has also been shown to improve disease-free survival after surgery in those patients who have residual cancer after receiving neoadjuvant chemotherapy in the KATHERINE trial [24]. Fam-trastuzumab was studied in a phase II clinical trial, which showed promising efficacy results in those patients diagnosed with metastatic HER2-positive breast cancer who failed T-DM1 [25,26]. Using monoclonal antibodies for cancer therapy is an effective and efficient strategy to treat breast cancer, but it has its own drawbacks such as the cost, treatment duration and frequency, resistance, and tolerance. Furthermore, these monoclonal antibodies show temporary disease control once the tumor is metastasized; hence, there is a need for therapies that elicit anti-tumor effects on metastatic tumors. Due to the aggressiveness of HER2-positive breast cancer, there is also a need to minimize the chance of relapse in those with a curable disease.

Despite the development of targeted therapies, tyrosine kinase inhibitors, as well as monoclonal antibodies and their toxin conjugates, all metastatic tumors develop resistance, and nearly one-third of HER2+ breast cancer patients develop resistance to all these therapies [7,27]. Thus, passive immunotherapy approaches have limitations and need continuous administration over a long period. On the other hand, a vaccine which introduces antigens acts on the cancer cells, causing prolonged activation of the immune system. Vaccines have a number of advantages compared to chemotherapy and monoclonal antibodies. Potential cancer relapse can be averted by activating long-term immunological memory with an effective vaccine that can protect against various tumor antigens. Vaccines are not required to be administered frequently and, historically, vaccines are comparatively safer than chemotherapy [28]. The first attempt to use a cancer vaccine was more than a century ago. In 1902, von Leyden and Blumenthal used an autologous tumor cell suspension as a vaccine and treatment for cancer patients [29]. During the 1950s, animal studies revealed that cancer tumors induced in mice by chemicals were immunogenic. Since then, there have been attempts to design a vaccine for cancer. Among breast cancer types, HER2-positive and triple-negative breast cancer (TNBC) subtypes are most immunogenic [30]. Thus, for these types of cancer, activating the patient’s immune system is a promising approach. Although overall progress is slow and clinical translation of this knowledge faced challenges, preclinical studies provided strong support for cancer vaccines, and there are some success stories.

Cancer vaccines can be broadly classified as preventive and therapeutic. Preventive vaccines are used to ward off any future infections, whereas therapeutic vaccines are used to treat a person with active disease [28]. Sipuleucel-T (Provenge) was the first therapeutic cancer vaccine approved by the FDA in the year 2010 for the treatment of metastatic castrate-resistant prostate cancer (mCRPC) [31]. Table 1 provides a list of FDA-approved preventive cancer vaccines. In this article, we will focus on therapeutic vaccines. Currently, a couple of therapeutic vaccines are approved by the FDA, which are listed in Table 2. Ongoing clinical trials of therapeutic vaccines are listed in Table 3. The success of these vaccines depends on several factors, including understanding the tumor microenvironment, strategies for reactivating the immune system utilizing different vaccine candidates, and vaccine formulations. Numerous review articles about cancer vaccines have been published over the past five years [30,32,33,34,35,36,37,38,39,40,41,42,43,44,45]. In this article, we provided details about the tumor environment, different types of vaccines, their advantages and disadvantages, and the current status of various vaccine candidates with a focus on vaccines for breast cancer.

## 2. Tumor Microenvironment and Its Modulation for Effective HER2 Vaccines

The human immune system is a complex network containing a variety of cells that effectively fight against pathogens and TAAs [46]. These TAAs are presented to the immune system in two ways; directly by the tumor cells and/or by antigen-presenting cells (APCs). In the process, the TAAs are degraded to immunogenic peptides and then presented to T cells via the major histocompatibility complex (MHC), eventually resulting in an immune response. However, this process is suppressed in the tumor microenvironment. The microenvironment around the tumor plays a significant role in its progression and control of cancer growth [47]. The tumor microenvironment is rich in molecules such as cyclooxygenase 2 (COX-2), vascular endothelial growth factor (VEGF), interleukin-6, interleukin-10, stem cell factor-1, macrophage-colony stimulating factor (M-CSF), and transforming growth factor (TGF-β) that are involved in the suppression of immune system functions and promote activation, invasion, and metastasis of tumors [48]. Furthermore, several mechanisms have been reported to be involved in immunosuppression, including expansion of myeloid-derived suppressor cells (MDSCs) [49], tumor-associated macrophages, and other myeloid cells [50,51], perturbation of cytokine networks [52], changes in host metabolism [53], and the production of amino acid-degrading enzymes and indoleamine 2,3-dioxygenase 1 (IDO1) [54]. Costimulatory signals such as B7, CD40, 4-1BBL [55], and OX40L [56] that are involved in the activation of T cells are absent in tumors of epithelial origin. Dendritic cells are also suppressed, resulting in a poor immune response against tumors [57]. These compounded immunological anomalies can lead to altered DC and T cell function and result in an impaired immune response against tumor cells [58]. The identification of the various causes for the immunosuppression and discovery of various molecules and TAAs (HER2, carbohydrate antigens, telomerase reverse transcriptase (hTERT), and mucin-1 (MUC-1)) led to the development of various strategies for the treatment of breast cancers by activating the immune system [46]. Research has demonstrated that patients with HER2-positive cancers have HER2 antibodies, prompting researchers to focus on a strategy to enhance patients’ immunity for the treatment of HER2-positive breast cancers [59]. Several strategies for developing vaccines against cancer are being investigated, including the use of peptides, proteins, APCs, tumor lysates, tumor cells, DNA, mRNA, and viral vectors [47].

## 3. Peptide-Based Cancer Vaccines

In the past decade, peptide-based vaccines have attracted a lot of attention for their potential use against cancer. There is a wealth of information about peptide-based cancer vaccine therapies in the literature [60,61,62]. Peptide-based cancer vaccines offer several possible advantages, including ease of synthesis, being cost-effective compared to other cancer-based vaccines, tolerable side effects, and safety. Additionally, computer-based algorithms can be applied while screening amino acid sequences for candidates with MHC class I-restricted peptide epitopes of the TAAs, and these candidates can be tested experimentally for their antigen-specific immune response.

T cell-based vaccines induce immune responses by delivering synthetic T cell epitopes into the body. T cell-based vaccines were originally studied to subsequently activate cytotoxic T lymphocytes (CTLs). Both CTLs and T helper cells were activated using short peptides; however, now, longer peptides are used to activate both CTLs and T helper cells. These peptides, when injected into a patient, bind to human leukocyte antigen (HLA) classes I and II of the APCs and form a peptide–HLA complex. This complex, when recognized by CTLs, is activated and proliferates. This results in an immune response, thereby attacking cancer cells [63].

On the other hand, B cell-based vaccines induce immune response via the B cell epitope of the specific TAA/tumor-specific antigen (TSA). In this type of vaccine, antibodies are produced and bind to the antigen of interest. The major advantage is MHC-I molecules are not involved in the generation of a response. Even if MHC-I molecules are downregulated by tumor evasion mechanisms, it will not affect the immune response against tumors [63].

The first clinical trials for the peptide-based vaccines were performed in the year 1990 by using a single epitope–peptide. E75 is a 9 amino acid-long peptide derived from the HER2 receptor and is predicted to bind HLA-A2, thus activating CTLs [64,65,66]. E75 is the most studied cancer vaccine. Several phase I studies were conducted by injecting peptide as a vaccine by mixing it with different immunoadjuvants. Results show that the vaccine is safe and is able to induce peptide-specific CTLs. Later, additional studies were evaluated by combining E75 with a granulocyte-macrophage colony-stimulating factor (GM-CSF) in 187 node-positive and high-risk node-negative breast cancer patients. Results concluded that the 5-year disease-free survival (DFS) was 89.7% for those who received E75 and 80.2% in those who received placebo, respectively. In phase III clinical trials, E75 with the adjuvant GM-CSF vaccine (Neuvax) was evaluated in patients with low HER2 expression (IHC 1+/2+). This combination was found to have no difference between placebo and Neuvax in DFS events resulting in the termination of clinical trials; however, future studies should be done combined with other medications [67].

GP2, an immunogenic peptide, a fragment of the transmembrane domain of HER2 (654–662), is a 9 amino acid-long peptide (IISAVVGIL) vaccine. It binds to the HLA-A2 molecule, but with lesser affinity compared to E75 [68], and activates CTLs. The phase I clinical trial suggested that GP2 with GM-CSF is safe and tolerated in patients with lymph node-negative breast cancer [68]. The phase II clinical trials were conducted in the clinically disease-free patients with node-positive and high-risk node HER2-expressing tumors (immunohistochemistry (IHC) 1+–3+). Results did not show a significant difference in response to the vaccine compared to the control groups in the rate of reoccurrence; however, it can be inferred from the trials that the vaccine is safe to be injected. Furthermore, there was a trend toward clinical significance in patients with HER2-overexpressed tumors [69].

AE37 is a peptide with 15 amino acids which activates CD4+ T helper cell (T_h_) lymphocytes [70]. In phase I clinical trials conducted on patients with different HER2-expressed breast cancer of all stages and IHC of 1+ to 3+, it was demonstrated that the vaccine has no significant effect on the DFS rate in patients with high HER2-expressing receptors on their breast tissue [69].

Limited research has been performed on B cell peptide vaccines. The success of trastuzumab as a therapeutic agent for breast cancer has led to an interest in B cell peptide vaccines. A phase I study was performed in metastatic breast cancer patients with three HER2 peptides derived from the HER2 receptor formulated with influenza virosomes. The study results showed that the vaccine is safe. In about 80% of the patients, it was found to be immunogenic. The antibodies developed in the patients can be compared to those of the current antibody-based HER2 treatment drugs [63,71]. Another phase I clinical trial for the vaccine containing two HER2 B cell epitomes that are binding sites for trastuzumab and pertuzumab was performed [72]. The aim of the vaccine is to overcome the resistance associated with trastuzumab and pertuzumab. This study was performed on 49 patients diagnosed with metastatic and/or recurrent solid tumors and showed that the vaccine is safe, elicits anti-tumor effects, and has the ability to overcome the resistance associated with trastuzumab and pertuzumab. Thus, this vaccine could be used as an alternative to monoclonal antibodies.

Even though peptide-based vaccines have several advantages, they do have some limitations. Peptide-based vaccines need a suitable adjuvant in order to produce an efficient immune response. The immune response is limited to a few epitopes, which results in a limited response against tumor cells. Other limitations include secondary structure, enzymatic stability, short half-life, and high rates of elimination [73,74,75]. There are examples of attempts to improve the balanced induction of both CD8 and CD4 T cells by using multivalent synthetic long peptides (SLPs) containing both MHC class I and class II epitopes [76].

## 4. Protein-Based Cancer Vaccines

While most of the attempts made using peptide-based vaccines have not shown a significant breakthrough compared to injecting a whole protein into the body, it has many theoretical advantages that may overcome the disadvantages associated with peptide-based vaccines. The major advantage of utilizing a whole protein (HER2 intra- or extracellular domains) as vaccines is that it contains both HLA class I and II epitopes; hence, specific HLA restrictions can be avoided. Long polypeptides or protein-based vaccines can significantly activate T cells resulting in a heightened immune response and superior T cell activation [77,78].

Unlike peptide-based vaccines, the prospect of utilizing protein-based vaccines has not been explored extensively. The first clinical study was performed with the HER2 intracellular domain (a fragment sequence from 676 to 1255 of the full-length HER2/neu) with the aim to evaluate whether the vaccine can generate immunogenicity. In this study, 29 patients who had HER2-positive breast or ovarian cancer and were in remission after traditional treatment were injected with different doses (25, 150, and 900 µg) of the vaccine. Results showed that the vaccine was well-tolerated, and HER2 ICD-specific T cell immunity developed in approximately 89% of the patients who completed the whole vaccine schedule. About 82% of the patients developed HER2/neu-specific immunoglobulin G antibody immunity. Furthermore, there were no reports of grade 2–4 toxic events [79]. Additionally HER-2/neu helper peptide based vaccines have been found to be effective in BC patients [80] A study was performed by Hamilton et al. [81] with the aim to evaluate immunogenicity, safety, and effect of the anti-HER2 protein. The vaccine, dHER2 [82], is a recombinant protein consisting of an extracellular domain (ECD) and a fragment of the intracellular domain (ICD) of HER2 combined with the adjuvant AS15. The twelve patients enrolled in the study with trastuzumab-refractory HER2-overexpressing metastatic breast cancer received the vaccine and oral lapatinib. Results indicated that all the patients in the study were prompted with the anti-HER2-specific antibody, and there were no reports of cardiotoxicity. Reports also showed the overall survival at 300 days was found to be 92% (95% CI: 77%–100%), suggesting a potential survival benefit in patients with HER2-overexpressing breast cancers refractory to trastuzumab [81].

## 5. Whole Cell-Based Vaccines

Most of the non-cell-based cancer vaccines are designed using a single tumor-associated antigen (TAA), and a major problem in developing vaccine therapies is the selection of an appropriate TAA that would maximize the immune response. Immunizing BC patients with tumor cells isolated from the patient can circumvent the problems associated with antigen selection. The principle behind this strategy is that a tumor cell harbors a wide variety of TAAs that would help in inducing a strong immune response. Tumor cells isolated from patients are used to develop autologous tumor cell-based vaccines (ATCVs). ATCVs consist of both characterized and uncharacterized TAAs that could help in launching a polyclonal response against a wide variety of tumor cells [83]. However, the process of developing ATCVs for individual patients is complex and expensive; hence, allogeneic tumor cell lines can be used as an alternative for the development of cell-based vaccines [84]. Whole cell-based vaccines can also be manipulated to express cytokines or chemokines to maximize the immune response against the injected whole-cell vaccine [85]. The addition of the granulocyte-macrophage colony-stimulating factor (GM-CSF) to a whole tumor cell vaccine stimulates the migration of DCs, T cells, eosinophils, and macrophages to the site of vaccination [85].

Two ongoing and three completed clinical trials have explored the efficacy of ATCVs in BC patients. In a completed study, 121 patients diagnosed with breast cancer, metastatic breast cancer, or ovarian cancer were vaccinated with an autologous breast tumor cell infected with Newcastle disease virus (NDV). The 4-year overall survival (OS) was 96%, thus validating the efficacy of the vaccine [86]. In a different study, 42 breast cancer patients were vaccinated with a vaccine mix consisting of autologous and allogenic breast tumor cells, three TAAs combined with GM-CSF and IL-2 [87]. Post-vaccination, a significant increase in lymphocyte proliferation was observed in 57–100% of the patients enrolled in the study [87]. Elliott et al. enrolled 37 breast cancer patients with suppressed immunity into a study and vaccinated them with a whole-cell vaccine consisting of autologous and allogenic tumor cells supplemented with adjuvants. Post-vaccination, it was observed that the 10-year survival of vaccinated patients with depressed immunity increased significantly compared to the historic controls of unvaccinated patients [88]. In the above three clinical studies, the whole cell-based vaccines were found to be safe and did not elicit any significant toxicity. Currently, two active clinical studies sponsored by the Dana–Faber Cancer Institute are in progress (NCT00317603, Vaccination with Autologous Breast Cancer Cells Engineered to Secrete Granulocyte-Macrophage Colony-Stimulating Factor (GM-CSF) in Metastatic Breast Cancer Patients, available at: clinicaltrials.gov; NCT00880464, Autologous Vaccination with Lethally Irradiated, Autologous Breast Cancer Cells Engineered to Secrete GM-CSF in women with Operable Breast Cancer, available at: clinicaltrials.gov). A detailed review of the clinical and preclinical studies related to ATCVs and breast cancer therapy can be found elsewhere [83]. The aforementioned clinical studies have demonstrated that ATCVs can be used as a highly effective and safe vaccine in BC patients. However, one disadvantage of using ATCVs is the high variability in the vaccine and the tedious vaccine manufacturing process [83].

ATCV manufacturing is dependent upon a patient’s specific tumor tissue. Therefore, it can be considered personalized medicine. The ATCVs may not have broad-spectrum application that is expected from a traditional vaccine. Allogenic vaccines are similar to ATCVs except that the source material is obtained from a different individual or from well-established cancer cell lines that are known to express specific TAAs [89]. A phase I clinical trial involving 28 metastatic BC patients was carried out to investigate the efficacy of a combination therapy using an allogeneic vaccine along with chemotherapy [90]. The allogeneic vaccine was formulated with TAAs obtained from two Her2/neu positive adenocarcinoma breast cancer cell lines SKBR3 and T47D. This vaccine was administered either alone or in combination with cyclophosphamide (CY) and doxorubicin (DOX) [91]. This study demonstrated that the vaccine alone or along with low-dose chemotherapy could induce an effective HER2-specific humoral and T cell-mediated immunity [89]. In another phase I study, an HLA-A2^+^-matched allogeneic MDA-MB-231 breast cancer cell line was transfected with the costimulatory molecule B7-1 (CD80) and used as a vaccine against stage IV BC [92]. Although no tumor regression was observed, the vaccinated patients did show an increase in tumor-specific immune activity [92]. The data available from clinical trials demonstrate the efficacy of cell-based allogenic vaccines in stimulating a measurable immune response [93,94]. The safety of an allogeneic vaccine was investigated in a clinical trial sponsored by Paul Ehrlich Institute, Langen, Germany (NCT01127074, Vaccination of Metastatic Breast Cancer Patients With a CD80-modified Allogeneic Cancer Cell Line (KS2422) (KS2422-vacc)) and in another clinical trial sponsored by Beth Israel Deaconess Medical Center (NCY00625755, A Phase I/II Study to Assess the Safety and Efficacy of Vaccinations With Allogenic Dendritic Cells: Autologous Tumor-Derived Cells Subjected to Electrofusion in Patients With AJCC Stage IV Renal Cell Carcinoma) [95]. However, a major problem with the development of allogeneic tumor cell vaccines is the use of cell lines that may not represent the actual antigen repertoire of the tumor.

## 6. Dendritic Cell-Based Vaccines

Dendritic cells (DCs) are highly specialized antigen-presenting cells that can process exogenous and endogenous antigens and present them to CD4^+^ T cells and CD8^+^ T cells, respectively [96,97]. DCs are the strongest modulators of primary immune response and can be exploited to generate highly effective DC-based vaccines [98]. Non-active or immature dendritic cells (iDCs) are usually isolated from the peripheral blood of cancer patients. The iDCs are then supplied with tumor-associated antigens (TAAs), recombinant DNA/RNA encoding tumor antigens, or DC/tumor hybrids [99]. The antigen-laden iDCs are then stimulated by exposure to specific cytokines for stimulation/maturation [100]. Stimulated/mature DCs are then infused back into patients wherein they present the cancer antigens to CD4^+^/CD8^+^ T cells, thus launching a robust anti-tumor T cell response [99]. Kugler et al. demonstrated the efficacy of a DC-based vaccine in patients suffering from advanced BC and ovarian cancer. Autologous DCs were pulsed with HER2/neu- or MUC1-derived peptides to generate a DC-based vaccine. Ten patients included in this pilot study showed a strong immunogenic response with no side effects [101]. The lack of side effects when autologous DCs are used for vaccine production can be exploited to generate potent DC-based vaccines for BC. Using an alternative approach, Avigan et al. fused patient-derived tumor cells with autologous DCs to generate fusion cells [102]. The fusion cell-based vaccine showed a strong anti-tumor response in patients suffering from metastatic BC and renal cancer [102]. The use of patient-derived tumor cells or cell lysates provides a wide variety of antigens to the immune system, thus facilitating a strong immunogenic response [103]. Zhang et al. generated a whole antigen vaccine against BC by fusing DCs with TNBC cells. The DC–TNBC hybrid was found to potentially elicit anti-tumor immunity by facilitating lymphocyte proliferation [104]. Preclinical studies for the development of DC-based vaccines for BC have shown some promising results. Sakai et al. modified DCs by transducing them with a non-signaling *neu* oncogene, which thwarted the growth of BC in BALB-neu transgenic mice [105]. A detailed explanation of the clinical studies related to DC-based vaccines has been discussed elsewhere [103].

## 7. DNA-Based Vaccines

Recently, the use of DNA-based vaccines has emerged as an effective vaccination strategy against cancer [106]. DNA vaccines have the potential to induce an antitumor immune response in breast cancer patients [107,108,109]. DNA vaccines are based on the dogma that the gene encoding a tumor antigen can be transfected and expressed in an APC. Physiologically, such antigens are further processed and presented to launch a strong and viable antitumor immune response. The most important aspects of DNA vaccination are the selection or design of a potent plasmid vector and an efficient delivery system coupled with monitoring of post-vaccination immune response. The plasmid used in DNA vaccines is usually of bacterial origin with CMV or a chimeric SV40–CMV promoter [110,111]. DNA-based vaccines are designed by using different types of TAAs. The TAAs are usually expressed exclusively in tumors or overexpressed by oncogenes. HER2/neu and mammaglobin-A (Mam-A) are oncoproteins that are overexpressed in breast cancer and have been used as target antigens in developing DNA vaccines. Norell et al. carried out a pilot clinical trial wherein eight patients suffering from advanced/metastatic breast cancer were administered a DNA vaccine containing signaling-deficient full-length version of HER2/neu along with low doses of IL-2 and GM-CSF. A strong humoral response was observed after HER2/neu vaccination, although no substantial improvement in the T cell response was elicited [112]. Mam-A is a 93 amino acid secretoglobin protein that is highly overexpressed in breast cancer and serves as an ideal target antigen. Kim et al. carried out a phase I clinical trial and administered a DNA vaccine carrying Mam-A cDNA to 15 Mam-A^+^ patients, and the post-vaccination immune response was monitored. After six months, the first seven patients enrolled in the study displayed an increase in ICOS^Hi^CD4^+^ T cells and a decrease in Foxp3C CD4C T cells [109]. The activated ICOS^Hi^CD4^+^ T cells expressed IFN-γ instead of IL-10 and were observed to cause preferential lysis of Mam-A-expressing breast cancer cells [113]. The present studies demonstrate the effectiveness of DNA vaccines in controlling breast cancer. However, the safety and the immunogenic mechanisms of DNA-based vaccines need to be further investigated.

## 8. Future Direction and Concluding Remarks

Breast cancer treatment using chemotherapy, hormonal therapy, passive immunotherapy, and other modalities has made a major contribution to the treatment of breast cancer. However, long-lasting effects are limited, and disease relapse and progression are observed in some patients. The discovery of breast cancer as immunogenic and the success of therapeutic vaccines such as Sipuleucel-T in treating prostate cancers raised the prospect of utilizing vaccination to manage breast cancer. Several preclinical studies are ongoing, and many vaccine candidates for treating breast cancers are currently in clinical trials. Some vaccine candidates in the advanced stage of clinical trials are showing promising results in treating breast cancer. The vaccine candidates for managing HER2-positive breast cancers are progressing well with promising results. A single-agent E75 peptide-based vaccine candidate is being studied in a phase III clinical trial and in combination with trastuzumab in a phase II study. Active immunotherapy could be an effective treatment regimen for managing breast cancer along with other therapies such as surgery, radiation, chemotherapy, endocrine therapy, and monoclonal antibodies. Active immunotherapy has the ability to produce antibodies for specific TAA, which promotes long-lasting effects. However, until now, no therapeutic vaccines have been approved by the US FDA for treating breast cancer. The success of cancer vaccines depends on a better understanding of the tumor microenvironment, including immune-suppressing pathways and tumor-evading pathways, the discovery of specific tumor-associated antigens, effective vaccine formulations, etc. There is promising efficacy data regarding the treatment of breast cancer by designing personalized vaccines based on TTAs and genetic mutations. In the case of personalized medicine, effective molecular stratification of breast cancer, vaccine formulation, and cost-effective vaccine manufacturing process need to be considered. In addition, clinical trials combining immunotherapy with other treatments that might produce an effective and synergic treatment regimen for breast cancer patients need to be explored.

While therapeutic cancer vaccines have shown some promise, they have not shown significant clinical benefits compared to immunotherapy such as immune checkpoint blockade. Hence, combination strategies with immune checkpoint inhibitors and antiangiogenic therapies have been proposed. Clinical trials consisting of large cohorts of patients are necessary to evaluate therapeutic efficacy of the proposed vaccine therapies [33]. Considering the cost of cancer drugs and the survival rate, mutation of proteins that are involved in cancer development, and resistance pathways, therapeutic vaccines have promise in the future of cancer therapy.

## Figures and Tables

**Table 1:** List of FDA-approved preventive cancer vaccines.

Name of the Vaccine	Cancer Type Prevented
Cervarix	HPV-related anal, cervical, head and neck, penile, vulvar, and vaginal cancers
Gardasil-4	HPV-related anal, cervical, head and neck, penile, vulvar, and vaginal cancers
Gardasil-9	HPV-related anal, cervical, head and neck, penile, vulvar, and vaginal cancers
Hepatitis B (HBV) vaccine (HEPLISAV-B)	HBV-related hepatocellular carcinoma

Cancer Vaccines: Preventive, Therapeutic, Personalized. Cancer Research Institute website. Updated January 2020. Accessed 15 October 2020 (https://www.cancerresearch.org/immunotherapy/treatment-types/cancer-vaccines).

**Table 2:** List of FDA-approved therapeutic cancer vaccines.

Therapeutic Cancer Vaccines	Cancer Type Treated
Bacillus Calmette–Guérin (BCG)	Early-stage bladder cancer (through local instillation into the bladder)
Sipuleucel-T (Provenge)	Prostate cancer

Cancer Vaccines: Preventive, Therapeutic, Personalized. Cancer Research Institute website. Updated January 2020. Accessed 15 October 2020 (https://www.cancerresearch.org/immunotherapy/treatment-types/cancer-vaccines).

**Table 3:** List of various cancer vaccines currently under clinical trials (clinicaltrials.gov).

	Name/Conditions	Interventions	Clinical Phase	Clinical Trial Identifier
1	Mammaglobin-A DNA Vaccine In Breast Cancer Patients Undergoing Neoadjuvant Endocrine Therapy	Mammaglobin-A DNA Vaccine	Phase 1	NCT02204098
2	Vaccine Therapy in Preventing Cancer Recurrence in Patients With Non-Metastatic, Node-Positive, HER2 Negative Breast Cancer That is in Remission	pUMVC3-IGFBP2-HER2- IGF1R Plasmid DNA Vaccine Sargramostim	Phase 1	NCT02780401
3	A Study to Evaluate Concurrent VRP-HER2 Vaccination and Pembrolizumab for Patients With Breast Cancer	VRP-HER2 Pembrolizumab	Phase 2	NCT03632941
4	HER2 Directed Dendritic Cell Vaccine During Neoadjuvant Therapy of HER2+Breast Cancer	Dendritic Cell Vaccine (DC1) Neoadjuvant Chemotherapy	Phase 1	NCT03387553
5	Vaccine Therapy in Treating Patients With HER2- Negative Stage III-IV Breast Cancer	CD105/Yb-1/SOX2/CDH3/ MDM2-polyepitope Plasmid DNA Vaccine	Phase 1	NCT02157051
6	HER2 Pulsed DC Vaccine to Prevent Recurrence of Invasive Breast Cancer Breast Cancer	HER2 pulsed Dendritic Cell Vaccine	Phase 1	NCT02063724
7	HER2 Pulsed DC Vaccine to Prevent Recurrence of Invasive Breast Cancer Post Neoadjuvant Chemotherapy Breast Cancer	HER2 pulsed Dendritic Cell Vaccine	Phase 1	NCT02061423
8	QUILT-3.013: Study of Ad5 [E1-, E2b-]-HER2/Neu Vaccine (ETBX-021) in Subjects With Unresectable Locally Advanced or Metastatic HER2-Expressing Breast Cancer Cancer	ETBX-021	Phase 1	NCT02751528
9	Xenogeneic HER2/Neu DNA Immunization for Patients With Metastatic and High-Risk Breast Cancer.	MAB HER 2 (HERCEPTIN)	Phase 1	NCT00393783
10	Immune Response and Potential Booster for Patients Who Have Received HER2-pulsed DC1	HER2 DC1 Vaccine	Phase 2	NCT03630809
11	A Vaccine (H2NVAC) Before Surgery for the Treatment of HER2-Expressing Ductal Carcinoma In Situ	Granulocyte-Macrophage Colony-Stimulating Factor Multi-epitope HER2 Peptide Vaccine H2NVAC	Phase 1	NCT04144023
12	A Phase I/II Trial of HER2/Neu Pulsed DC1 Vaccine Combined With Trastuzumab for Patients With DCIS Breast Cancer	HER2 pulsed DC1 Drug: trastuzumab Drug: pertuzumab	Phase 1 Phase 2	NCT02336984
13	Vaccine Therapy With Sargramostim (GM-CSF) in Treating Patients With HER2 Positive Stage III-IV Breast Cancer or Ovarian Cancer	pNGVL3-hICD vaccine Biological: sargramostim	Phase 1	NCT00436254
14	TPIV100 and Sargramostim for the Treatment of HER2 Positive, Stage II-III Breast Cancer in Patients With Residual Disease After Chemotherapy and Surgery	Pertuzumab Sargramostim Trastuzumab Trastuzumab Emtansine Vaccine Therapy	Phase 2	NCT04197687
15	Vaccine Therapy in Treating Patients With Stage IV HLA-A2 and HER2 Positive Breast or Ovarian Cancer Receiving Trastuzumab	HER2/neu Peptide Vaccine	Phase 1 Phase 2	NCT00194714
16	Vaccine to Prevent Recurrence in Patients With HER2 Positive Breast Cancer	DC1 Vaccine WOKVAC Vaccine	Phase 2	NCT03384914
17	Phase II Trial of Combination Immunotherapy With NeuVax and Trastuzumab in High-risk HER2+ Breast Cancer Patients	NeuVax vaccine Drug: Trastuzumab Drug: GM-CSF	Phase 2	NCT02297698
18	Folate Receptor Alpha Peptide Vaccine With GM-CSF in Patients With Triple-Negative Breast Cancer	Low dose FR# vaccine Drug: Cyclophosphamide High dose FR# vaccine	Phase 2	NCT02593227

## Data Availability

Not applicable.

## Abbreviations

ADCC	antibody-dependent cell-mediated cytotoxicity
APCs	antigen-presenting cells
ATCVs	autologous tumor cell-based vaccines
BC	breast cancer
CMV	cytomegalovirus
COX-2	cyclooxygenase 2
CTL	cytotoxic T lymphocytes
CY	cyclophosphamide
DCs	dendritic cells
DOX	doxorubicin
ECD	extracellular domain
EGFR	epidermal growth factor receptor
ER	estrogen receptor
FDA	Food and Drug Administration
GM-CSF	macrophage colony-stimulating factor
HER	human epidermal growth factor receptor
HLA	human leukocyte antigen
hTERT	telomerase reverse transcriptase
ICD	intracellular domain
ICOS	inducible T-cell costimulator
iDCs	immature dendritic cells
IDO1	indoleamine 2,3-dioxygenase 1
IFN-γ	interferon gamma
IHC	immunohistochemistry
mCRPC	metastatic castrate-resistant prostate cancer
M-CSF	macrophage-colony stimulating factor
MDSCs	myeloid-derived suppressor cells
MHC	major histocompatibility complex
mRNA	messenger RNA
MUC-1	mucin-1
PR	progesterone receptor
SLPs	synthetic long peptides
TAA	tumor-associated antigens
T-DM1	trastuzumab emtansine
TGF	transforming growth factor
TNBC	triple-negative breast cancer
TSA	tumor-specific antigen
VEGF	vascular endothelial growth factor
WHO	World Health Organization
